# *BIN1* rs744373 SNP and *APOE* alleles specifically associate to common diseases

**DOI:** 10.3389/frdem.2022.1001113

**Published:** 2022-10-28

**Authors:** Maria Cachide, Liliana Carvalho, Ilka Martins Rosa, Jens Wiltfang, Ana Gabriela Henriques, Odete A. B. da Cruz e Silva

**Affiliations:** ^1^Neurosciences and Signalling Group, Medical Sciences Department, Institute of Biomedicine - iBiMED, University of Aveiro, Aveiro, Portugal; ^2^Department of Psychiatry and Psychotherapy, University Medical Centre Goettingen (UMG), Georg-August University, Goettingen, Germany; ^3^German Centre for Neurodegenerative Diseases (DZNE), Goettingen, Germany

**Keywords:** amphiphysin 2, AD biomarkers, dementia, diabetes mellitus, dyslipidemia, respiratory diseases

## Abstract

*APOE* ε4 and *BIN1* are the two main genetic risk factors for sporadic Alzheimer's Disease (AD). Among several *BIN1* variants, the rs744373 is frequently associated with AD risk by contributing to tau pathology and poor cognitive performance. This study addressed the association of *APOE* and *BIN1* rs744373 to specific characteristics in a Portuguese primary care-based study group, denoted pcb-Cohort. The study included 590 participants from five primary care health centers in the Aveiro district of Portugal. Individuals were evaluated and scored for cognitive and clinical characteristics, and blood samples were collected from the volunteers meeting the inclusion and exclusion criteria (*N* = 505). *APOE* and *BIN1* genotypes were determined, and their association with cognitive characteristics and other diseases that might contribute to cognitive deficits, namely depression, hypertension, type 2 diabetes, dyslipidemia, osteoarticular diseases, gastrointestinal diseases, cardiovascular and respiratory diseases, was assessed. The diseases attributed to the study group were those previously diagnosed and confirmed by specialists. The results generated through multivariate analysis show that *APOE* ε4 carriers significantly associated with poorer cognitive performance (OR = 2.527; *p* = 0.031). Additionally, there was a significant risk of dyslipidemia for *APOE* ε4 carriers (OR = 1.804; *p* = 0.036), whereas *BIN1* rs744373 risk-allele carriers were at a significantly lower risk of having dyslipidemia (OR = 0.558; *p* = 0.006). Correlations were evident for respiratory diseases in which *APOE* ε4 showed a protective tendency (OR = 0.515; *p* = 0.088), and *BIN1* had a significative protective profile (OR = 0.556; *p* = 0.026). Not of statistical significance, *APOE* ε2 showed a trend to protect against type 2 diabetes (OR = 0.342; *p* = 0.093), in contrast *BIN1* rs744373 risk-allele carriers were more likely to exhibit the disease (OR = 1.491; *p* = 0.099). The data here presented clearly show, for the first time, that the two top genetic risk factors for sporadic AD impact a similar group of common diseases, namely dyslipidemia, respiratory diseases, and type 2 diabetes.

## Introduction

Alzheimer's Disease (AD) is the world's most common type of dementia. It is a progressive neurodegenerative disease characterized by the impairment of several cognitive functions, namely emotional and social changes, and deficits in memory, attention, concentration and language (Balasa et al., 2011).

Late-Onset Alzheimer's Disease (LOAD) represents nearly 95% of AD cases (Bertram and Tanzi, 2004), affecting individuals aged 65 or older. The scientific community accepts that genes play a crucial role in disease onset and development, thus contributing to LOAD complexity (Bertram and Tanzi, 2004). The gene encoding apolipoprotein E (*APOE*), located on chromosome 19, has been consistently linked to LOAD, and the ε4 allele is considered the most significant risk factor for this dementia (Bertram and Tanzi, 2012). Decreased amyloid-β (Aβ) clearance (Zlokovic, 2013) and increased Aβ aggregation (Bertram and Tanzi, 2004) were more prevalent in ε4 carriers than in non-carriers. Furthermore, Aβ load and senile plaque accumulation (one of the hallmarks of the disease) strongly correlate to *APOE* ε4 dosage at autopsy (Rebeck et al., 1993; Schmechel et al., 1993). On the other hand, the ε2 allele has a protective effect against the onset and development of AD (Bertram and Tanzi, 2012).

Over the years, several Genome-Wide Association Studies (GWAS) have identified more than 20 loci linked to LOAD risk (Lambert et al., 2009, 2013). Among these is the Bridging Integrator 1 (*BIN1*), considered the second most significant genetic risk factor for sporadic AD (Kunkle et al., 2019). *BIN1* is a gene associated with endocytic pathways. Thus, likely to be involved in amyloid-β protein precursor (AβPP) metabolism and Aβ production, strengthening the relevance of endocytic mechanisms in AD etiology and progression (Itoh and De Camilli, 2006), alongside phosphorylation-related processes (Gandy et al., 1993). Furthermore, *BIN1* has a potential role in regulating the actin cytoskeleton and might interact with microtubule-associated proteins like tau, whose dysregulation can result in neurofibrillary tangles (NFTs), another hallmark of AD (Itoh and De Camilli, 2006). The SNP rs744373 is the most commonly reported *BIN1* variant conferring AD risk, with an Odds Ratio (OR) of 1.17–1.19 and a global frequency close to 40% (Antúnez et al., 2011; Hu et al., 2011; Almeida et al., 2018). This variant has been correlated with the rate of cognitive decline and AD progression (Franzmeier et al., 2021), increasing tau loads and contributing to poor cognitive performance and tau-related memory deficits (Franzmeier et al., 2019). Additionally, a study showed an association between *BIN1* rs744373 and high levels of total tau and tau protein phosphorylated at threonine 181, measured in CSF samples of mild cognitive impairment (MCI) and AD patients (Wang et al., 2016).

In addition to genetic factors, other age-related diseases are prevalent among the elderly population and contribute to cognitive decline (Duthie et al., 2011). Epidemiological and molecular studies suggest that common disorders such as depression (DEP) (Novais and Starkstein, 2015), cardiovascular diseases (CVD) and cardiovascular risk factors, among them type 2 diabetes (DM), hypertension (HYP) and dyslipidemia (DYS) are associated with increased dementia risk (Tini et al., 2020). Growing evidence also supports an association between cognitive impairment and respiratory diseases (RESP) (Villeneuve et al., 2012; Singh et al., 2014; Liao et al., 2015), osteoarticular diseases (OA) (Weber et al., 2019) and gastrointestinal diseases (GID) (Rosa et al., 2017). Inflammation is a crucial mechanism underlying the association between dementia and these age-related disorders (Santiago and Potashkin, 2021). Likewise, cerebrovascular disease is commonly observed in AD patients and also associates with DM, DEP, and DYS (Santiago and Potashkin, 2021). Thus, cerebrovascular damage might be another link between these age-related diseases and dementia.

In the context of other diseases, studies show that *APOE* increases the risk of DEP (Wang et al., 2019). Consistently, individuals with CVD or other cardiovascular-related risk factors such as HYP and DM are more prone to AD if they carry the *APOE* ε4 allele (Peila et al., 2002; Kang et al., 2005). Contrastingly, besides AD, *BIN1* has not been associated with most of the abovementioned age-related diseases. Two studies evaluated the *BIN1* association with DM, but the results are contradictory (Greenbaum et al., 2016; Vacínová et al., 2017). The interplay between other diseases and dementia needs further clarification, and more studies need to address these associations and how *APOE* and *BIN1* might influence these pathologies.

Given the high incidence of dementia worldwide, in which a high percentage is attributed to LOAD cases, it is imperative to readdress the highest AD risk genes in the context of cognitive deficits and other diseases. Therefore, in this study, we investigate the frequency of *APOE* alleles in a primary care-based group (pcb-Cohort) involving 590 Portuguese participants from five randomly chosen primary health care centers in the Aveiro district of Portugal. To evaluate the cognitive deficits as normal, to moderate or severe, we performed the Clinical Dementia Rate (CDR) on the study population. Likewise, the relevance of the rs744373 variant of *BIN1* as a potential risk factor associated with cognitive deficits in this Portuguese population was investigated. Finally, the possible associations of these two risk loci with HYP, DYS, OA, CVD, DEP, GID, DM, and RESP, were addressed in the context of the abovementioned association with dementia.

This study provides insights into population-specific risk factors, reinforcing *APOE* ε4 as a risk factor to cognitive deficits among the Portuguese population. Although preliminary and requiring further replications, our findings support that the two top genetic risk factors for AD affect similar age-related pathologies that could contribute to dementia.

## Materials and methods

### Study design

A cross-sectional population-based survey on a primary care-based Portuguese volunteer group of 590 individuals (pcb-Cohort) was carried out as previously described (Rosa et al., 2017). For this study, five primary health care centers, in the Aveiro district of Portugal, were randomly selected. In brief, participants completed a structured interview covering their respective lifestyles, and clinical history was collected. Next, cognitive evaluations and dementia screening tests, namely CDR, Mini-Mental State Examination (MMSE), the Geriatric Depression Scale (GDS), the Katz Activities Daily Living (ADL), and Instrumental Activities Daily Living (IADL), were performed on all 590 volunteers.

Clinical data from the study participants, compiled by physicians and health professionals, was accessed via collaboration with the medical staff at all sites and was thoroughly investigated. Clinical data available from clinical records were scored, such as information regarding the presence of other diseases, namely HYP, DYS, OA, CVD, DEP, GID, DM, and RESP. HYP was diagnosed after observing persistent elevation of systolic blood pressure (SBP) ≥140 mmHg and/or diastolic blood pressure (DBP) ≥90 mmHg in several temporally distinct measurements. Similarly, DYS was diagnosed by measuring total cholesterol, HDL cholesterol, and triglycerides after a 12-h fasting period, with repeated analyses at a minimum interval of 4 weeks. DEP was diagnosed according to the Diagnostic and Statistical Manual of Mental Disorders (DSM-5) criteria, in which the individual must have at least five symptoms of either a depressed mood or loss of interest or pleasure for 2 weeks. In the present study, CVD included cardio pathologies, cardio arrhythmias, myocardial infarction, acute coronary syndrome, coronary revascularization or other arterial revascularization procedure, ischemic stroke and peripheral arterial disease. GID comprised dyspepsia, esophagitis, gastritis, duodenitis, inflammatory bowel diseases, diverticulosis, diverticulitis and anusitis. Finally, RESP included the following chronic pathologies: allergic rhinitis, asthma, chronic obstructive pulmonary disease, restrictive pathologies, and sarcoidosis. For RESP, pathologies considered acute, self-limited, or infectious, such as tonsillitis and pneumonia, were excluded. All common diseases considered in the present study were scored positive based on the previous diagnosis with confirmation by a specialist in the reference hospital for the Aveiro Region. Common diseases unable to be confirmed were not included when scoring for the prevalence of the different pathologies among the study participants.

A total of 568 volunteers fulfilled the inclusion criteria and were processed for *APOE* allele and *BIN1* rs744373 SNP genotyping. Procedures regarding this phase are described below.

### Blood collection and genotyping

**Figure 1:**
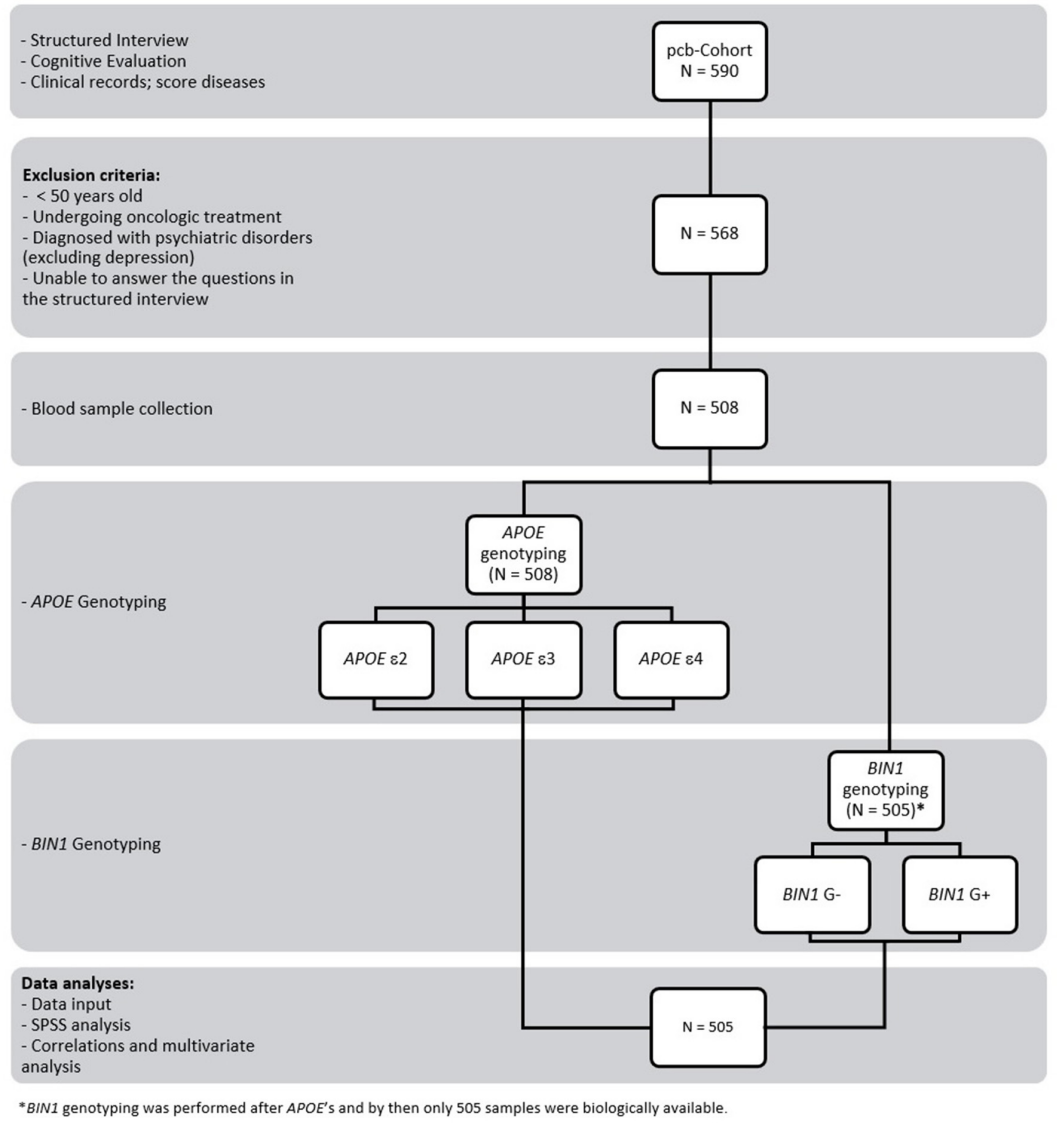
Study design and workflow. The study involved 590 volunteers who were subjected to a structured interview and cognitive evaluation. Clinical data were also collected, and the diseases of each participant were scored. A total of 568 individuals met the inclusion and exclusion criteria, although it was possible to collect blood from only 508. *APOE* genotyping was performed before *BIN1*. Meanwhile, 3 samples were no longer biologically available for genotyping (*BIN1* population = 505). Therefore, only 505 samples (available for both *APOE* and *BIN1*) were considered for the present study. Data input and subsequent analyses were carried out using SPSS.

For each volunteer, blood was collected into 3 tubes for whole blood, serum, and plasma (3+5+5 ml, respectively), according to standard procedures. Samples were immediately aliquoted and frozen at −80°C. Whole blood samples collected in EDTA tubes were available for genotyping of *APOE* for only 508 individuals (Figure 1). *APOE* genotyping was performed prior to *BIN1*. Meanwhile, three samples were no longer biologically available for genotyping (*BIN1* population = 505). Therefore, only 505 samples (available for both *APOE* and *BIN1*) were considered for the present study.

*APOE* and *BIN1* genotyping were carried out by direct blood PCR using a modified Phusion Hot Start II High-Fidelity DNA Polymerase protocol (Phusion Blood Direct PCR Master Mix; Thermo Fisher Scientific). For *APOE*, genetic polymorphic regions were amplified using 1–2 μl of blood and the primers *APOE*-Fw 5'-CGGGCACGGCTGTCCAAGGAG-3' and *APOE*-Rev 5'-CACGCGGCCCTGTTCCACCAG-3', yielding a fragment of around 300-bp, as previously described (Rosa et al., 2017). The PCR conditions were as follows: 98°C for 5 min; 35 cycles of 98°C for 1 s, 64°C for 5 s, and 72°C for 15 s; and a final extension step at 72°C for 1 min. For *BIN1* (rs744373), PCR of the polymorphic regions was carried out using 2 μL of whole blood from each patient, 2 × Phusion Blood Direct Master Mix (Thermo Fisher Scientific), and 0.5 μM gene-specific primer *BIN1*-forward 5'- AAGACGGAGAGAGGAGGCAT-3' and *BIN1*-reverse 5'-CCATCTTCTTCTGCTCTCCCA G−3 ′, yielding a fragment of around 767-bp. The PCR conditions were: 98°C for 5 min; 35 cycles of 94°C for 1 min, 63°C for 30 s, and 72°C for 46 s; and a final extension step at 72°C for 5 min. Afterwards, PCR products were purified with sodium acetate (3M, pH 5.2), and Sanger sequencing was performed. Results were analyzed to determine the nucleotide polymorphisms and the respective *APOE* and *BIN1* genotypes. For the study design, the number of volunteers genotyped was 505, which is a reasonable sample size given the population density of the Aveiro district (Schulz and Grimes, 2005; Rosa et al., 2017).

### Statistical analysis

Analyses of the data collected at each phase were carried out, blind to the data from the other stages, using the Statistical Package for the Social Sciences (SPSS) version 26 (Marôco, 2021). Categorical variables were assessed through the examination of frequencies. In contrast, continuous variables were evaluated by the generation of descriptive methods (means, standard deviations) to investigate the differences in the group (CDR, cognitive performance, depression groups, *APOE* allele carriers, *BIN1* G+ carriers vs. normal groups).

Regarding the multivariate analysis, logistic regression was used for the dichotomous dependent variables (risk allele G of *BIN1* rs744373 variant and *APOE* carriers of ε2 or ε4), considering the socio-demographic and cognitive characteristics, as well as the diseases scored for each volunteer. The reference group included non-carriers of the risk allele; that is, for *APOE* ε4, the reference group was the one that lacked this allele; for *APOE* ε2, the reference group was the one that did not have this allele; and for *BIN1* the reference group was the one that did not have the risk allele G. The Odds Ratio was calculated. This ratio, if >1, indicates a risk factor, and if < 1 indicates a protective factor. In the present study, this methodology was used to identify the risk/protective factors concerning both *BIN1* and *APOE*.

A two-sided statistical test was carried out for each analysis, and a *p*-value < 0.05 was considered statistically significant. *P*-values between 0.05 < and < 0.1 were considered a trend.

## Results

### *APOE* and *BIN1* frequencies in the pcb-Cohort

**Figure 2:**
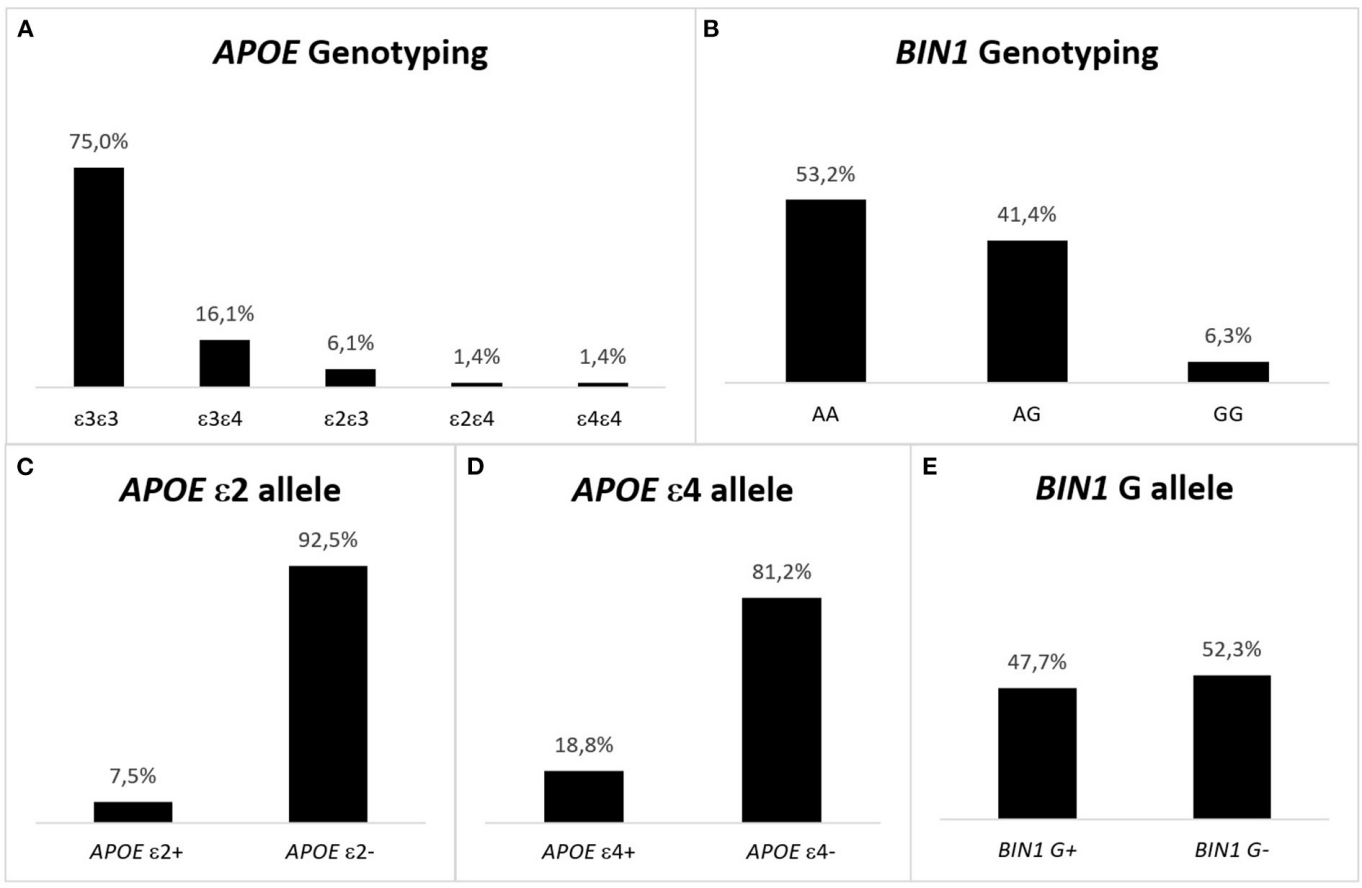
Representation of the genotype and allelic frequencies for *APOE* and *BIN1* in the pcb-Cohort. **(A)** *APOE* haplotype in the pcb-Cohort. The most frequent haplotype is ε3ε3, while ε2ε2 individuals are absent. The prevalence of ε4ε4 is 1.4%. ε4 is the risk allele of *APOE*; being a carrier of the allele confers risk for developing AD. This risk is higher when two ε4 alleles are present. **(B)** *BIN1* genotype in the pcb-Cohort. The most frequent genotype is AA (53.2%), while GG cases are the least frequent (6.3%). G is the risk allele of *BIN1* rs744373; being a carrier of the allele confers risk for developing AD. This risk is higher when two G alleles are present. **(C)** *APOE* ε2 alleles in the pcb- Cohort. 38 (7.5%) individuals have the ε2 allele of *APOE* (protective for AD). **(D)** *APOE* ε4 alleles in the pcb- Cohort. There are 95 (18.8%) individuals with the ε4 allele of *APOE* (risk allele for AD). **(E)** *BIN1* G alleles in the pcb-Cohort: 241 (47.7%) of the volunteers of the pcb-Cohort are carriers of the G allele of the *BIN1* SNP rs744373 (risk allele for developing AD).

In the study population, the most predominant *APOE* haplotype was the ε3ε3, representing 75% of the individuals, followed by ε3ε4 (16.1%) and ε2ε3 (6.1%). It is noteworthy that ε2 and ε4 have opposite effects, but in the pcb-Cohort only 1.4% of the participants had the ε2ε4 haplotype (Figure 2). Similarly, only 1.4% of cases were ε4ε4, being one of the least prevalent haplotypes. As for ε2ε2 haplotype, it was absent in the study population. Regarding *BIN1*, the most prevalent genotype was the AA (53.2%), followed by AG (41.4%) and the GG genotype, representing only 6.3% of the individuals.

A similar analysis by alleles shows that only 7.5% of the study population had the protective ε2 allele of *APOE*, while more than twice as many individuals (18.8%) carried the allele that confers risk of developing AD (allele ε4). In turn, the G allele of the *BIN* rs744373 variant, which confers risk of developing LOAD, was present in almost half of the study population (47.7%).

### Socio-demographic and cognitive characteristics of the pcb-Cohort as a function of *APOE* and *BIN1* SNP rs744373

Relevant characteristics, namely socio-demographic and cognitive deficits within the pcb-Cohort and associations with *APOE* and *BIN1* rs744373, were analyzed and are summarized in Table 1. Overall, no significant associations were evident for ε3 carriers (data not shown). In contrast, there is a skewed prevalence for the stratified genotypes among these characteristics, where *APOE* ε4 carriers associated with cognitive deficits, while *APOE* ε2 and *BIN* only associated with age.

**Table 1:** *APOE* and *BIN1* allele associations with the social-demographic and cognitive characteristics of the pcb-Cohort.

**Socio-demographic and** **cognitive characteristics**		**pcb-Cohort** ***N* = 505 (%)**					
			***APOE***		***p*-value**	***APOE***		***p*-value**	***BIN1***		***p*-value**
			**ε4–**	**ε4+**		**ε2–**	**ε2+**		**G –**	**G +**	
			***N* = 410 (%)**	***N* = 95 (%)**		***N* = 467 (%)**	***N* = 38 (%)**		***N* = 264 (%)**	***N* = 241 (%)**	
Gender	Male	150 (29.7%)	118^a^ (28.8%)	32^a^ (33.7%)	0.346	142^a^ (30.4%)	8^a^ (21.1%)	0.225	81^a^ (30.7%)	69^a^ (28.6%)	0.614
	Female	355 (70.3%)	292^a^ (71.2%)	63^a^ (66.3%)		325^a^ (69.6%)	30^a^ (78.9%)		183^a^ (69.3%)	172^a^ (71.4%)	
Age group	< 65 years	175 (34.7%)	144^a^ (35.1%)	31^a^ (32.6%)	0.888	155^a^ (33.2%)	20^b^ (52.6%)	0.015*	85^a^ (32.2%)	90^a^ (37.3%)	0.446
	≥65 years	330 (65.3%)	266^a^ (64.9%)	64^a^ (67.4%)		312^a^ (66.8%)	18^b^ (47.4%)		179^a^ (67.8%)	151^a^ (62.7%)	
	Mean age ± SD^⋎^	67.9 ± 9.2	67.8 ± 9.2	68.2 ± 9.0	0.676	68.1 ± 9.1	65.4 ± 9.6	0.088	68.7 ± 8.8	67.0 ± 9.6	0.034*
CDR	CDR = 0	254 (50.3%)	214^a^ (52.2%)	40^a^ (42.1%)	0.038*	231^a^ (49.5%)	23^a^ (60.5%)	0.382	135^a^ (51.1%)	119^a^ (49.4%)	0.193
	CDR = 0.5	187 (37.0%)	151^a^ (36.8%)	36^a^ (37.9%)		175^a^ (37.5%)	12^a^ (31.6%)		90^a^ (34.1%)	97^a^ (40.2%)	
	CDR ≥ 1	64 (12.7%)	45^a^ (11.0%)	19^b^ (20.0%)		61^a^ (13.1%)	3^a^ (7.9%)		39^a^ (14.8%)	25^a^ (10.4%)	
	Mean CDR Sum Box ± SD^⋎^	1.3 ± 2.9	1.1 ± 2.8	1.8 ± 3.3	0.042*	1.3 ± 3.0	0.7 ± 1.3	0.234	1.4 ± 3.1	1.1 ± 2.6	0.296
MMSE	MMSE +	46 (9.1%)	36^a^ (8.7%)	10^a^ (10.4%)	0.606	43^a^ (9.10%)	3^a^ (7.9%)	0.796	24^a^ (9.1%)	22^a^ (9.1%)	0.988
	Mean MMSE ± SD^⋎^	27.5 ± 3.5	27.6 ± 3.5	27.1 ± 3.7	0.190	27.5 ± 3.6	27.2 ± 2.8	0.605	27.5 ± 3.6	27.5 ± 3.4	0.970
GDS	GDS +	157 (31.1%)	119^a^ (29.0%)	38^b^ (40.0%)	0.037*	147^a^ (31.5%)	10^a^ (26.3%)	0.509	79^a^ (29.9%)	78^a^ (32.4%)	0.554
	Mean GDS ± SD^⋎^	3.5 ± 3.3	3.4 ± 3.2	4.0 ± 3.5	0.119	3.5 ± 3.3	3.2 ± 3.0	0.620	3.4 ± 3.3	3.5 ± 3.2	0.740
ADL—Dependent		29 (5.7%)	20^a^ (4.9%)	9^a^ (9.5%)	0.083	27^a^ (5.8%)	2^a^ (5.3%)	0.895	16 ^a^ (6.1%)	13^a^ (5.4%)	0.748
IADL—Dependent		161 (31.9%)	126^a^ (30.7%)	35^a^ (36.8%)	0.250	151^a^ (32.3%)	10^a^ (26.3%)	0.444	84^a^ (31.8%)	77^a^ (32.0%)	0.975

Data are presented as n (%) and % is expressed as a function of the total in each column (pcb-Cohort, n = 505; *APOE*ε4–, n = 410; *APOE*ε4+, n = 95; *APOE*ε2–, n = 467; *APOE*ε2+, n = 38; BIN1 G–, n = 264; BIN1 G+, n = 241) or mean ± standard deviation (SD). Statistical test used: Chi square (χ^2^) test; (^⋎^) Student t-Test. CDR, Clinical Dementia Rate; MMSE, Mini Mental State Examination adapted for the Portuguese population (Rosa et al., 2018); GDS, Geriatric Depression Scale; ADL, Activities of Daily Living; IADL, Instrumental Activities of Daily Living; *APOE*, apolipoprotein E; ε-allele of *APOE*; BIN1, Bridging Integrator 1; G allele of BIN1 polymorphism rs744373; (+) with the risk allele. All 505 individuals were evaluated for MMSE, GDS, ADL, and IADL; however, only the positive and dependent cases are represented, respectively. ^a, b^, The same superscript letter denotes a subset of categories whose column proportions do not differ significantly from each other at the 0.05 level. Different superscript letters denote column proportions that differ significantly from each other at the 0.05 level. In bold and with *p-value < 0.05. *P*-values > 0.05 and < 0.10 are underlined.

In the pcb-Cohort, women were more prevalent (70.3%). Our results do not show any significant gender effect associated with either *APOE* ε4 (*p* = 0.346), *APOE* ε2 (*p* = 0.225), or BIN1 (*p* = 0.614). Although not significative (*p* = 0.088), our results also showed a decrease in the mean age between non-carriers (68.1 years) and carriers of the *APOE* ε2 allele (65.4 years). Moreover, in the age group < 65 years ε2 carriers were more frequent than non-carriers (52.6% vs. 33.2%), while in the age group ≥ 65 years ε2 carriers were less frequent than non-carriers (47.4% vs. 66.8%), reaching statistical significance (*p* = 0.015). Regarding *BIN1*, there was a significant decrease (*p* = 0.034) in the mean age of individuals with the G allele (67.0 years) compared to non-carriers (68.7 years). However, when grouping individuals by age (< 65 years and ≥65 years), there were no significant differences in the frequency of the *BIN1* risk allele (*p* = 0.446).

An association between *APOE* ε2 and cognitive features of the study population was not observed. In contrast, the frequency of individuals with moderate to severe cognitive deficits based on the CDR scores (CDR ≥ 1) was more significant in ε4 carriers (*p* = 0.038), and the mean in CDR scores was also significantly higher in carriers (1.8 score) compared to non-carriers (1.1 score). Regarding the GDS scale (GDS ≥ 5), we observed an association between *APOE* ε4 carriers and high GDS scores (*p* = 0.037). Additionally, our results showed a higher association trend between the ε4 allele of *APOE* and ADL (*p* = 0.083), with an increase in ADL-dependent cases among ε4 carriers. No associations between *APOE* and neither the MMSE nor the IADL were found.

Regarding *BIN1*, the frequency of the G risk allele increased in individuals with CDR scores equal to 0.5 (34.1% in non-carriers compared to 40.2% in carriers), contrary to the other two groups (CDR = 0 and CDR ≥ 1). Still, this result was not significant (*p* = 0.193). Furthermore, no significant associations with the remaining cognitive tests were identified.

### Logistic regression of *APOE* and *BIN1* in the Portuguese pcb-Cohort

Regarding socio-demographic characteristics, our results showed no association between age and *APOE* ε4 or *BIN1*. Nevertheless, a lower association trend between age and *APOE* ε2 when comparing non-carriers to carriers (Table 2) was observed. The group of individuals over 65 years old showed 47% reduced odds of having the ε2 allele compared to the younger age group (% of 1–0.53; OR = 0.534; 95% CI 0.256–1.112; *p* = 0.094).

**Table 2:** Logistic regression of *APOE* and *BIN1* alleles in the pcb-Cohort.

	***APOE*** ε**4**+			***APOE*** ε**2**+			***BIN1*** **G**+		
	**OR**	**95% CI**	***p*-value**	**OR**	**95% CI**	***p*-value**	**OR**	**95% CI**	***p*-value**
*APOE* ε4+	–	–	–	1.154	0.468–2.844	0.756	1.690	1.054–2.711	0.029*
*APOE* ε2+	1.069	0.430–2.655	0.887	–	–	–	1.872	0.919–3.815	0.084
*BIN1* G+	1.693	1.051–2.728	0.030*	1.844	0.898–3.786	0.096	–	–	–
Female	0.728	0.418–1.270	0.264	1.528	0.609–3.837	0.367	1.156	0.738–1.809	0.527
Age ≥65	0.939	0.557–1.584	0.814	0.534	0.256–1.112	0.094	0.918	0.614–1.373	0.677
HYP	0.708	0.415–1.208	0.205	1.000	0.470–2.123	0.999	0.888	0.585–1.349	0.579
DYS	1.804	1.040–3.129	0.036*	0.550	0.254–1.194	0.131	0.558	0.367–0.847	0.006**
OA	1.276	0.771–2.111	0.344	1.048	0.496–2.213	0.903	0.876	0.590–1.299	0.510
CVD	1.357	0.757–2.432	0.305	0.808	0.351–1.858	0.615	1.319	0.842–2.067	0.226
DEP	0.822	0.479–1.412	0.479	0.893	0.410–1.945	0.776	1.231	0.811–1.867	0.328
GID	0.826	0.459–1.485	0.522	1.633	0.721–3.701	0.240	0.860	0.550–1.347	0.511
DM	0.816	0.437–1.525	0.524	0.342	0.098–1.195	0.093	1.491	0.928–2.398	0.099
RESP	0.515	0.240–1.105	0.088	0.870	0.311–2.433	0.790	0.556	0.331–0.934	0.026*
CDR = 0.5	1.211	0.704–2.084	0.490	0.652	0.291–1.460	0.298	1.277	0.842–1.937	0.250
CDR≥1	2.527	1.089–5.865	0.031*	0.574	0.112–2.955	0.507	0.583	0.278–1.221	0.153
MMSE	0.576	0.214–1.554	0.276	1.224	0.253–5.927	0.802	1.346	0.597–3.031	0.474
GDS	1.649	0.974–2.791	0.063	0.780	0.338–1.801	0.561	1.097	0.716–1.678	0.671
ADL	1.692	0.595–4.814	0.324	1.418	0.236–8.508	0.703	0.847	0.339–2.116	0.723
IADL	0.908	0.516–1.599	0.739	1.032	0.417–2.556	0.945	1.083	0.691–1.699	0.728

The reference category for *APOE* ε4+ is *APOE* ε4–, or absence of the allele; for *APOE* ε2+ the reference category is *APOE* ε2–; and for *BIN1* G+ the reference category is *BIN1* G–. –, this parameter is set to zero because it is redundant. For diseases, the reference category is the absence of the disease. For the other categories, the reference is as indicated in the previous tables, i.e., male or female. OR, Odds Ratio: risk or protective value for each parameter studied; 95% CI, 95% Confidence Interval of the OR. HYP, Hypertension; DYS, Dyslipidemia; OA, Osteoarticular disease; CVD, Cardiovascular disease; DEP, Depression; GID, Gastrointestinal disease; DM, Type 2 Diabetes Mellitus; RESP, Respiratory diseases. CDR, Clinical Dementia Rate; MMSE, Mini Mental State Examination adapted for the Portuguese population (Rosa et al., 2018); GDS, Geriatric Depression Scale; ADL, Activities of Daily Living; IADL, Instrumental Activities of Daily Living; *APOE*, apolipoprotein E; ε-allele of *APOE*; *BIN1*, Bridging Integrator 1; G allele of *BIN1* polymorphism rs744373; (+) with the risk allele. In bold and with **p-value < 0.01. In bold and with *p-value < 0.05. *P*-values > 0.05 and < 0.10 are underlined.

As for the cognitive characteristics, our results showed an association between *APOE* ε4 and cognitive deficits (Table 2). Carriers of the ε4 allele were 2.5 times more likely to have a CDR score above 1 (OR = 2.527; CI 95% = 1.089–5.865; *p* = 0.031). Likewise, our results showed a tendency for a higher association between depressive states and a positive GDS score among ε4 carriers (OR = 1.649; CI 95% = 0.974–2.791; *p* = 0.063).

The multivariate analysis of the studied diseases showed results aligned with the bivariate analyses (Supplementary Table 1). DYS was statistically significant among the studied diseases for both *APOE* ε4 and *BIN1*. *APOE* ε4 to carriers were 1.8 times more likely to have DYS (OR = 1.804; CI 95% = 1.040–3.129; *p* = 0.036) than non-carriers. Likewise, carriers of the *BIN1* G allele had a 44% reduced risk of having DYS compared to non-carriers (% of 1–0.56; OR = 0.558; 95% CI 0.367–0.847; *p* = 0.006).

Our results also showed that carriers of the G allele of *BIN1* had a 44% reduced risk of having RESP compared to non-carriers (% of 1–0.56; OR = 0.556; 95% CI 0.331–0.934; *p* = 0.026). On the contrary, although not reaching statistical significance, there was a 48% reduced risk of having RESP diseases among carriers of the ε4 allele of *APOE*, compared to non-carriers (% of 1–0.52; OR = 0.515; 95% CI 0.240–1.105; *p* = 0.088).

DM was another disease showing a trend to be associated with both *APOE* and *BIN1*. Despite not reaching statistical significance, individuals with the ε2 allele had a 66% reduced risk of having DM compared to non-carriers (% of 1–0.34; OR = 0.342; 95% CI 0.098–1.195; *p* = 0.093). Conversely, when comparing carriers of the risk allele G of *BIN1* rs744373 to non-carriers, the first showed a higher association trend with a greater risk of having DM (OR = 1.491; CI 95% = 0.928–2.398; *p* = 0.099).

Interestingly, *APOE* and *BIN1* seem to influence each other. According to the logistic regression, the presence of the *APOE* ε4 allele significantly increased by 1.7 times the odd of having the G allele of *BIN1* rs744373 (*p* = 0.030) and vice versa (*p* = 0.029). Moreover, without reaching statistical significance, we also observed a similar trend for a higher association between *APOE* ε2 and BIN1.

## Discussion

Regarding *APOE* alleles, the results of the present study are consistent with other findings, reporting a higher frequency of the ε3 allele and ε3ε3 haplotype, and a lower frequency of *APOE* ε2 (Davignon et al., 1988; Mahley, 1988; Farrer et al., 1997; Smith, 2000). Remarkably, we observed a high frequency of the G allele of *BIN1* SNP rs744373 in the study population, reinforcing that it is one of the most relevant LOAD risk genes, particularly this variant.

### *APOE* and *BIN1* rs744373 and socio-demographic and cognitive characteristics

In the pcb-Cohort, our results show a higher percentage of ε2 carriers in the age group < 65 years and a lower rate in the age group ≥65 years, compared to non-carriers (*p* = 0.015). Data suggest that younger generations have a higher frequency of this allele than older generations. *APOE* ε2 has been associated with longevity (Shinohara et al., 2020). However, contrary to our results, previous studies showed higher frequencies of *APOE* ε2 in elderly individuals and centenarians compared to younger populations (Cauley et al., 1993; Sebastiani et al., 2019). Nevertheless, it is essential to mention that the *APOE* ε2 variant represents the most recent variant of this risk gene (Fullerton et al., 2000), suggesting that selective pressures contributed to the evolution and global distribution of human *APOE* alleles over time (Huebbe and Rimbach, 2017). Thus, this preliminary observation may relate to genetic variations of the younger population. Still, more comprehensive studies need to be carried out in this respect, particularly in larger cohorts, to evaluate potential generational effects on allele frequencies. Of note, *APOE* ε2 is not only a protective gene but might also increase the risk of certain cerebrovascular diseases and neurological disorders (Li et al., 2020), which could also contribute to the reduced frequency of *APOE* ε2 carriers among ≥65 years old individuals in our study population.

Further, *APOE* ε4 carriers significantly associated with cognitive deficits (*p* = 0.038), contrary to ε2 carriers (*p* = 0.382). In fact, *APOE* ε4 emerged as a significant risk factor, increasing the susceptibility to develop dementia (OR = 2.527; *p* = 0.031). This result is in line with previous reports where *APOE* ε4 was associated with poor cognitive performance even in healthy individuals (Caselli, 2009; Wisdom et al., 2011). Other studies also reported that the ε4 variant increases the risk of developing AD, while the ε2 variant reduces AD risk (Loy et al., 2014). Moreover, in the present study, while executing the CDR test, *APOE* ε4 carriers presented worse memory performance and other cognitive alterations such as diminished orientation, judgment, and problem-solving skills. This result indicates that the ε4 allele correlates with memory problems and changes in a broader range of cognitive functions, corroborating published data (Yasuno et al., 2012). This conclusion was further strengthened by observing a potential association between the presence of the ε4 allele and ADL-dependent individuals (*p* = 0.083). Therefore, in the study population, *APOE* ε4 proved to be a risk factor for the existence of cognitive deficits, contributing to doubling the risk of having a score of CDR >1 (OR = 2.527; *p* = 0.031), often associated with dementia. Nevertheless, early identification and management of dementia in the primary care setting remain a challenge (Parmar et al., 2014), and dementia is often underdiagnosed by clinicians in primary care centers and underreported by patients and families (Amjad et al., 2018). Due to dementia-related symptoms being considered part of the normal aging process (Schulz and Grimes, 2005), an estimated 50% of primary care patients 65 years or older are not diagnosed with this syndrome (Iliffe et al., 2009). Thus, underdiagnosed dementia might contribute to the doubled risk of CDR≥1 among the *APOE* ε4 carriers' group, as was observed in this study group.

In our study population, *APOE* ε4 allele was also associated with GDS (*p* = 0.037), the test evaluating depression. In the multivariate analysis, we observed a trend showing that ε4 carriers are more likely to have a depressed state (OR = 1.649; *p* = 0.063). This observation is in line with previous studies (Wang et al., 2019). Reports have suggested that amyloid-associated depression may precede the onset of AD, particularly in *APOE* ε4 carriers (Sun et al., 2008; Qiu et al., 2016). Moreover, previous research reports that the ε4 allele may increase the likelihood of depression by about 4 times in women (Delano-Wood et al., 2008). In contrast, men do not show an association between DEP and this *APOE* allele (Delano-Wood et al., 2008). Other studies report no association between depressive states and *APOE* ε4 carriers (Locke et al., 2013). Thus, more research should be conducted on the interplay between DEP and this risk gene.

Regarding *BIN1*, in the pcb-Cohort the G risk allele of rs744373 SNP does not seem to be associated with either socio-demographic characteristics or cognitive deficits (Table 1). Thus, we could not replicate Seshadri et al. (2010) results, which showed a significant association between that variant and AD cases. Still, it is crucial to remember that in such an intricate and complex disease as AD, it becomes more challenging to replicate GWAS discoveries due to the heterogeneity of different populations. More studies are required to evaluate the role of this and other *BIN1* variants in larger populations with different characteristics, to have a greater insight into the relationship between this risk gene and cognitive deficits that might result in dementia.

### *APOE* and *BIN1* rs744373 in the context of other diseases

In the pcb-Cohort, both the ε4 allele of *APOE* and *BIN1* rs744373 are significantly associated with DYS. *APOE* ε4 allele considerably increased the risk of having DYS (OR = 1.804; *p* = 0.036). On the contrary, *BIN1* rs744374 had a protective profile and is likely to prevent the disease by 50%, compared to non-carriers of the G allele (OR = 0.558; *p* = 0.006). Several studies have demonstrated associations between the different *APOE* haplotypes and plasma levels of lipids and lipoproteins (Sing and Davignon, 1985; Boerwinkle and Utermann, 1988; Mooijaart et al., 2006). *APOE* ε4 carriers usually have increased total cholesterol levels and low density protein (LDL)-cholesterol (Sun et al., 2014). Also, numerous common genetic variants have a combined effect on influencing plasma levels of HDL cholesterol (Spirin et al., 2007). Previous research suggested that higher plasma *APOE* and high-density lipoproteins (HDL) from early life might preserve cognitive functions in later life, especially in *APOE* ε4 carriers (Yasuno et al., 2012). A longitudinal study showed that *APOE* ε4 non-carriers with AD, have cholesterol metabolism dysfunction and functional harm with raised HDL-cholesterol levels, possibly due to lower availability of lipids to neuronal membranes (de Oliveira et al., 2017). Over the years, researchers have explored the interplay between DYS and dementia, however, the role of cholesterol in AD is still debatable. A study reported that protective variants of *APOE* against risk of AD also slow cognitive decline in patients with dementia, regardless of cholesterol variations, while therapy with lipophilic statins might benefit carriers of specific genetic variants (de Oliveira et al., 2020). The same was observed regarding two other protective variants, particularly among *APOE* ε4 carriers with AD (De Oliveira et al., 2022). To our knowledge, this is the first time that an association between *BIN1* rs744373 and DYS has been reported. Further studies should elucidate the nature of this association and how important it might be for dementia.

Given that *APOE* ε4 and *BIN1* are considered risk factors for AD and that previous studies have shown an association between respiratory diseases such as COPD and dementia (Villeneuve et al., 2012; Singh et al., 2014; Liao et al., 2015), one would expect that carriers of these risk variants would be more prone to RESP. Our data indicate a low association trend between *APOE* genotype and RESP diseases (*p* = 0.088). However, contrary to what we expected, the ε4 allele might decrease the likelihood of RESP (OR = 0.515). Similarly, *BIN1* seems to be a protective factor for RESP as the G allele significantly reduces the risk of having RESP (OR = 0.556; *p* = 0.026). Further studies should address the mechanism by which *APOE* ε4 or *BIN1* might influence the pathogenesis of RESP.

Our results also support a strong trend of *APOE* ε2 allele as a protective factor toward DM, with a reduction of type 2 diabetes odds by 66% in carriers of that allele (OR = 0.342; *p* = 0.093). Diabetic individuals have an increased risk of developing AD (Biessels et al., 2006), thus ε2 carriers may be less prone to both DM and AD. In a primary care setting, the screening and control of type 2 diabetes may aid in dementia prevention. Nevertheless, future studies in larger populations need to validate these observations.

Surprisingly, a higher association trend between the risk allele of *BIN1* SNP rs744374 and DM was also detected (*p* = 0.099). It is important to emphasize that in the present study, while *BIN1* showed a protective profile regarding DYS and RESP, in the case of DM, the rs744373 variant seems to be a risk factor (OR = 1.491), which also differs from the results of *APOE* ε2 (OR = 0.342). A previous study by Vacínová et al. (2017) reported no association between DM and the *BIN1* SNP rs744373. Further, a recent study showed that, among ≥65 years old subjects with DM, the rs6733839 variant of *BIN1* may contribute to individual changes in episodic memory performance (Greenbaum et al., 2016). Besides DM increasing AD risk by 2-fold (Mayeux and Stern, 2012), it is also a potential changeable risk factor for developing this type of dementia. Therefore, it might be altered or strengthened by other risk factors such as genetic causes (Lindenberger et al., 2008) and influenced by *BIN1*. Despite these findings, additional large-scale genetic studies in different populations are required to unravel the possible roles of *BIN1* in the overlap between the two pathologies. Also, future studies should explore if the coexistence of the rs744373 variant and DM could contribute to cognitive deficits.

Overall, the present study represents an essential step in elucidating genomic contributions to dementia and how LOAD's two top risk genes might be associated with other diseases influencing the onset and development of cognitive impairment. There are, nonetheless, some limitations. First, the small sample size of this study may be underpowered to detect the minor effects of genetic variants. Likewise, since it was beyond this study's scope, we did not address gene-gene or gene-environment interactions, which would be an asset given that cognitive performance is multifactorial. Further, we were not able to detect minor effects/confounding factors.

For these reasons, and as mentioned above, it is imperative to reproduce and validate these results in other cohorts with larger populations and investigate the possible association of other *BIN1* polymorphisms with cognitive deficits.

## Conclusion

To our knowledge, this was the first genetic study addressing the impact on cognitive deficits of both *APOE* and *BIN1* rs744373 on several diseases in a Portuguese population selected from a primary health care setting. Thus, it represents an important step in elucidating genomic contributions to cognitive deficits, offering some insights into population-specific risk factors.

As expected, *APOE* ε4 was a significant risk factor for cognitive deficits in the pcb-Cohort. Although rs744373 (*BIN1*) was not associated with an increased risk of cognitive deficits in this Portuguese population, we did not address other SNPs. Future studies in the Portuguese population should evaluate other *BIN1* variants in the context of cognitive deficits.

The present study showed a strong association between the two top genetic AD risk factors (*APOE* and *BIN1*) and other age-related pathologies such as DM, RESP, and DYS. Nevertheless, it is imperative to study their presence individually and profiles of coexisting diseases and risk genes to find new therapies and ways to prevent dementia, including AD.

## Data availability statement

The raw data supporting the conclusions of this article will be made available by the authors, without undue reservation.

## Ethics statement

The studies involving human participants were reviewed and approved by Ethics Committee for Health of the Central Regional Administration of Coimbra (CES da ARS Centro, protocol No. 012804-04.04.2012), Portuguese National Committee for Data Protection (Authorization N° 369/2012). The patients/participants provided their written informed consent to participate in this study.

## Author contributions

OCS, AH, and JW obtained funding. MC, LC, IR, OCS, and AH conceptualized and designed the study. IR collected cognitive and clinical data. LC, IR, and AH collected and processed blood samples. MC and LC performed the genotyping procedures. MC and IR analyzed data. MC, IR, AH, and OCS wrote and revised the paper. JW revised the paper. All authors read and approved the final manuscript.

## Funding

This work was funded by PdC no: 1811255, SAICT-45-2021-02 Portugal2020, FEDER, and also supported by PTDC/DTPPIC/5587/2014 and POCI-01-0145-FEDER-016904, Instituto de Biomedicina (iBiMED)-UIDB/04501/2020 and POCI-01-0145-FEDER-007628, the Fundação para a Ciĉncia e Tecnologia (FCT) of the Ministério da Educação e Ciência, COMPETE program, the QREN and the European Union (Fundo Europeu de Desenvolvimento Regional), by the Integrated Programme of SR&TD “pAGE” (CENTRO-01-0145-FEDER-000003), co-funded by Centro 2020 program, Portugal 2020, European Union, through the European Regional Development Fund and by MEDISIS – CENTRO-01-0246-FEDER-000018, Centro 2020 program, Portugal 2020, European Union. MC was supported by the FCT through an individual Ph.D. scholarship SFRH/BD/132995/2017.

## Conflict of interest

The authors declare that the research was conducted in the absence of any commercial or financial relationships that could be construed as a potential conflict of interest.

## Publisher's note

All claims expressed in this article are solely those of the authors and do not necessarily represent those of their affiliated organizations, or those of the publisher, the editors and the reviewers. Any product that may be evaluated in this article, or claim that may be made by its manufacturer, is not guaranteed or endorsed by the publisher.
