# Supplementary material for: BIN1 rs744373 SNP and APOE alleles specifically associate to common diseases
Source: Front Dement. 2022 Oct 28;1:1001113. doi: 10.3389/frdem.2022.1001113 (PMC11285651; doi:10.3389/frdem.2022.1001113)
Supplement: Supplementary file 1 [file Table_1.DOCX]

Supplementary Material

Supplementary Table 1 - APOE and BIN1 allele associations with common diseases of the pcb-cohort.

| **Common Diseases** | **pcb-cohort**  **N = 505 (%)** | | ***APOE*** | | | | **p-value** | | ***APOE*** | | | | **p-value** | | ***BIN1*** | | | | **p-value** | |  |
| --- | --- | --- | --- | --- | --- | --- | --- | --- | --- | --- | --- | --- | --- | --- | --- | --- | --- | --- | --- | --- | --- |
|  |  |  | **Ԑ4–**  **N = 410 (%)** | | **Ԑ4+**  **N = 95 (%)** | |  |  | **Ԑ2–**  **N = 467 (%)** | | **Ԑ2+**  **N = 38 (%)** | |  |  | **G –**  **N = 264 (%)** | | **G +**  **N = 241 (%)** | |  |  |  |
| **HYP** | | 310 (61.4%) | | 255a (62.2%) | | 55^a^ (57.9%) | | 0.438 | | 290^a^ (62.1%) | | 20^a^ (52.6%) | | 0.249 | | 165^a^ (62.5%) | | 145^a^ (60.2%) | | 0.591 | |
| **DYS** | | 300 (59.4%) | | 234^a^ (57.1%) | | 66^b^ (69.5%) | | **0.027*** | | 285^a^ (61.0%) | | 15^b^ (39.5%) | | **0.009**** | | 170^a^ (64.4%) | | 130^b^ (53.9%) | | **0.017*** | |
| **OA** | | 266 (52.7%) | | 214^a^ (52.2%) | | 52^a^ (54.7%) | | 0.655 | | 246^a^ (52.7%) | | 20^a^ (52.6%) | | 0.996 | | 143^a^ (54.2%) | | 123^a^ (51.0%) | | 0.482 | |
| **CVD** | | 276 (54.7%) | | 218^a^ (53.2%) | | 58^a^ (61.1%) | | 0.164 | | 261^a^ (55.9%) | | 15^a^ (39.5%) | | 0.051 | | 145^a^ (54.9%) | | 131^a^ (54.4%) | | 0.898 | |
| **DEP** | | 130 (25.7%) | | 130^a^ (31.7%) | | 29^a^ (30.5%) | | 0.823 | | 146^a^ (31.3%) | | 13^a^ (34.2%) | | 0.707 | | 78^a^ (29.5%) | | 81^a^ (33.6%) | | 0.326 | |
| **GID** | | 254 (50.3%) | | 108^a^ (26.3%) | | 22^a^ (23.2%) | | 0.523 | | 119 ^a^(25.5%) | | 11^a^ (28.9%) | | 0.638 | | 71^a^ (26.9%) | | 59^a^ (24.5%) | | 0.536 | |
| **DM** | | 100 (19.8%) | | 83^a^ (20.2%) | | 17^a^ (17.9%) | | 0.605 | | 97^a^ (20.8%) | | 3^a^ (7.9%) | | 0.055 | | 49^a^ (18.6%) | | 51^a^ (21.2%) | | 0.464 | |
| **RESP** | | 81 (16.0%) | | 72^a^ (17.6%) | | 9^a^ (9.5%) | | 0.053 | | 76^a^ (16.3%) | | 5^a^ (13.2%) | | 0.615 | | 52^a^ (19.7%) | | 29^b^ (12.0%) | | **0.019*** | |

Data are presented as n(%) and % is expressed as a function of the total in each column (pcb-Cohort, n = 505; *APOEε4-,* n = 410*; APOEε4+,* n = 95*; APOEε2-,* n = 467; *APOEε2+,* n = 38; *BIN1 G-,* n = 264; *BIN1 G+,* n = 241). Statistical test used: Chi square (χ^2^) test. APOE, apolipoprotein E; ε-allele of APOE; BIN1, Bridging Integrator 1; G allele of BIN1 polymorphism rs744373; (+) with the risk allele. HYP, Hypertension; DYS, Dyslipidemia; OA, Osteoarticular disease; CVD, Cardiovascular disease; DEP, Depression; GID, Gastrointestinal disease; DM, Type 2 Diabetes Mellitus; RESP, Respiratory diseases. All 505 individuals were evaluated for the common diseases, however, only the positive cases are represented. ^a, b^, The same superscript letter denotes a subset of categories whose column proportions do not differ significantly from each other at the 0.05 level. In bold and with **, p-value<0.01. In bold and with *, p-value<0.05. P values > 0.05 and < 0.10 are underlined.
